# An Evaluation of DNA Methyltransferase 1 (DNMT1) Single Nucleotide Polymorphisms and Chemotherapy-Associated Cognitive Impairment: A Prospective, Longitudinal Study

**DOI:** 10.1038/s41598-019-51203-y

**Published:** 2019-10-10

**Authors:** Alexandre Chan, Angie Yeo, Maung Shwe, Chia Jie Tan, Koon Mian Foo, Pat Chu, Chiea Chuen Khor, Han Kiat Ho

**Affiliations:** 1 Department of Pharmacy, National University of Singapore, Singapore, Singapore; 2 Department of Pharmacy, National Cancer Centre Singapore, Singapore, Singapore; 3 Duke-N.U.S. Graduate Medical School Singapore, Singapore, Singapore; 4 Department of Pharmacy, K.K. Women’s and Children’s Hospital, Singapore, Singapore; 5 Singapore Cord Blood Bank, Singapore, Singapore; 6 Human Genetics, Genome Institute of Singapore, Singapore, Singapore; 7 Singapore Eye Research Institute, Singapore, Singapore

**Keywords:** Neurological manifestations, Predictive markers

## Abstract

Strong evidence suggests that genetic variations in DNA methyltransferases (DNMTs) may alter the downstream expression and DNA methylation patterns of neuronal genes and influence cognition. This study investigates the association between a *DNMT1* polymorphism, rs2162560, and chemotherapy-associated cognitive impairment (CACI) in a cohort of breast cancer patients. This is a prospective, longitudinal cohort study. From 2011 to 2017, 351 early-stage breast cancer patients receiving chemotherapy were assessed at baseline, the midpoint, and the end of chemotherapy. DNA was extracted from whole blood, and genotyping was performed using Sanger sequencing. Patients’ self-perceived cognitive function and cognitive performance were assessed at three different time points using FACT-Cog (v.3) and a neuropsychological battery, respectively. The association between *DNMT1* rs2162560 and cognitive function was evaluated using logistic regression analyses. Overall, 33.3% of the patients reported impairment relative to baseline in one or more cognitive domains. Cognitive impairment was observed in various objective cognitive domains, with incidences ranging from 7.2% to 36.9%. The *DNMT1* rs2162560 A allele was observed in 21.8% of patients and this was associated with lower odds of self-reported cognitive decline in the concentration (OR = 0.45, 95% CI: 0.25–0.82, *P* = 0.01) and functional interference (OR = 0.48, 95% CI: 0.24–0.95, *P* = 0.03) domains. No significant association was observed between *DNMT1* rs2162560 and objective cognitive impairment. This is the first study to show a significant association between the *DNMT1* rs2162560 polymorphism and CACI. Our data suggest that epigenetic processes could contribute to CACI, and further studies are needed to validate these findings.

## Introduction

Chemotherapy-associated cognitive impairment (CACI) is highly prevalent among cancer patients receiving chemotherapy^1–3^. Reports have shown that CACI negatively affects patients’ social functioning and quality of life^3,4^. The exact mechanism underlying CACI has yet to be elucidated. However, genetic factors are known to contribute to CACI^5–8^.

Epigenetic modifications such as DNA methylation can modify intermediate neuronal gene expression, leading to changes in cognitive performance^9^. DNA methylation is catalyzed by DNA methyltransferase (DNMT) enzymes and involves the addition of a methyl group to the 5′-position of cytosine bases, primarily at cytosine-phosphate-guanine (CpG) dinucleoside sites, which results in gene silencing^10^. In mammalian cells, *DNMT1* is the most abundant form of DNA methyltransferases. It is primarily responsible for maintaining methylation and has a higher affinity for hemimethylated DNA^11^. DNMT-mediated DNA methylation regulates multiple aspects of neuronal development and function, with important roles in learning and memory^12–14^. Clinically, the dysregulated expression or aberrant function of *DNMT1* could affect neurocognitive function by altering the methylation patterns of cognitive gene targets^15,16^. Recently, a prospective study reported cognitive decline is associated with DNA methylation of leukocytes in breast cancer patients receiving chemotherapy, providing evidence of epigenetic links to CACI^17^.

The extent of methylation is controlled by the expression and function of *DNMT1*, which can be altered by single nucleotide polymorphisms (SNPs) situated within its genetic code^18^. *DNMT* SNPs have been studied in Alzheimer’s disease and schizophrenia^19,20^. However, *DNMT* SNPs have not been investigated in CACI. Using a candidate gene approach, we identified the *DNMT1* SNP rs2162560 from the literature^19,20^ and evaluated its association with CACI in a cohort of breast cancer patients. We hypothesize that carriers of the A allele of the rs2162560 polymorphism have increased DNA methylation activity that protects them from CACI.

## Methods

### Study design

This multicenter, prospective, longitudinal cohort study was conducted in Singapore between 2011 and 2017. This study was approved by the SingHealth Centralized Institutional Review Board. Written informed consent was obtained from all participants. All experiments were performed in accordance with relevant guidelines and regulations.

### Participants

Patients were eligible to participate in this study if they fulfilled the following inclusion criteria: (i) at least 21 years old, (ii) diagnosed with stage I-IIIA breast cancer, (iii) scheduled for four cycles of anthracycline- or taxane-based chemotherapy, (iv) no history of chemotherapy or radiotherapy, and (v) read and understand English or Mandarin. Patients who were (i) symptomatically ill, (ii) diagnosed with brain metastasis or any neuropsychiatric illness that may cause cognitive impairment, or (iii) physically or mentally incapable of giving written informed consent were excluded.

### Study procedure

The first time point (T1) occurred at baseline before the initiation of chemotherapy. The second time point (T2) was timed at 6 weeks after T1 and coincided with the first day of the third cycle of chemotherapy. The third time point (T3) was 12 weeks after T1 when the primary chemotherapy was completed. Overall, the approximate duration between each time point was 6 weeks. Patients completed both objective and subjective neuropsychological assessments and self-reported questionnaires to assess their health-related quality of life, fatigue, and anxiety. All tools were available in English and Chinese and were administered by trained bilingual interviewers.

### Assessment tools

Subjective cognitive functioning was assessed using the Functional Assessment of Cancer Therapy – Cognitive Function (FACT-Cog) version 3, which is a patient-reported questionnaire that measures self-perceived cognitive impairment^21^. It evaluates the cognitive domains of mental acuity, concentration, memory, functional interference, verbal fluency, and multitasking. The domain scores are calculated by summing the individual domain items, and the FACT-Cog summation score is obtained by adding all item scores together. The English and Chinese versions of the FACT-Cog used in this study were previously validated and have demonstrated equivalence and reliability^22^.

Objective cognitive function was initially assessed using Headminder. As Headminder was commercially discontinued in 2014, the Cambridge Neuropsychological Test Automated Battery (CANTAB, Cambridge Cognition Ltd., UK) was used for objective cognitive assessment for the remainder of the study. Both neuropsychological tests are language-independent and computer-based. For both batteries, four cognitive domains were assessed: processing speed, response speed, memory, and attention. The specific measures used by Headminder and CANTAB as neuropsychological assessments are described in Supplementary Table 1. These tests have been validated and show sensitivity in capturing alterations in neuropsychological performance in the four cognitive domains^23,24^.

Anxiety, cancer-related fatigue, and insomnia are known confounders of cognition and were assessed by validated patient-reported questionnaires – the Beck Anxiety Inventory (BAI)^25^, the Brief Fatigue Inventory (BFI)^26^, and the European Organization for Research and Treatment of Cancer Quality of Life Questionnaire C-30 (EORTC QLQ-C30)^27^, respectively.

### Defining cognitive impairment

The overall impairment in self-perceived cognitive function was defined as a reduction in the FACT-Cog summation score by ≥10.6 points during (T2) or after chemotherapy (T3) compared with the baseline value. This reduction is based on the minimal clinically important difference (MCID) that we previously established as clinically significant^28^. The FACT-Cog MCID range is in line with a change of 5–10% in the EORTC-QLQ-C30 scales, which is proposed to be the minimal clinically significant change. For individual cognitive domains, patients were considered to be experiencing clinically significant cognitive impairment if their domain scores at T2 or T3 were ≥15% lower than their baseline score^8^.

Reliable change index (RCI) scores were calculated to assess changes in objective cognition. RCI was computed by calculating the difference of Headminder or CANTAB scores between baseline and T2 or T3, subtracting the mean practice effect extrapolated from a normative group and dividing by the standard error of difference. The RCI for both Headminder and CANTAB were calculated using two separate reference groups (Supplementary Table 2). Patients were classified as having impairment in each of the cognitive domains if the RCI score was ≤−1.5^29^.

### Genotyping

Upon recruitment, 10 mL of whole blood was drawn from each participant into an ethylenediaminetetraacetic acid (EDTA) tube. The samples were centrifuged at 2500 rpm for 10 minutes within 30 minutes of collection. The buffy coat was drawn and stored at −80 °C until analysis. Genomic DNA was extracted from the buffy coat using the QIAamp DNA Blood Mini Kit (Qiagen) according to the manufacturer’s protocol. The regions containing polymorphisms were amplified by polymerase chain reaction (PCR) using the following optimized primers: 5′-AAGCACAAAGGCAGGTTCGC-3′ (forward) and 5′-GTGCCCAGCTGCAAAGTGTT-3′ (reverse). Genotyping was performed without knowledge of clinical outcomes by AITbiotech Pte Ltd. (Singapore).

### Statistical analysis

Descriptive statistics were used to summarize the demographic and clinical characteristics of the patients. Differences in the demographic data and questionnaire scores between patients with and without cognitive impairment were compared using the independent-sample t-test if normally distributed. The Mann-Whitney U-test was used to compare non-normally distributed continuous and ordinal data. The chi-squared test was used to identify differences in categorical demographic data between the two groups. The Friedman test was used to evaluate changes in questionnaire and neuropsychological test scores across the three time points.

Genotype and allele frequencies were checked for deviation from the Hardy-Weinberg equilibrium using the chi-squared test with one degree of freedom. Binary logistic regression analysis assuming a dominant model was carried out to evaluate associations between the SNP of interest and cognitive function while adjusting for ethnicity and documented confounders of CACI (age, fatigue, menopausal status, chemotherapy regimen, and education level)^30,31^. In addition, anxiety, insomnia and brain-derived neurotrophic factor (BDNF) rs6265 polymorphism status were incorporated as confounders in the analysis model for subjective cognitive impairment as they are associated with self-reported cognitive impairment in breast cancer patients^3,8^. Univariate analysis was conducted and any variables that achieved *P* ≤ 0.1 were included in the logistic regression model. Anxiety (total BAI score), fatigue (total BFI score), and insomnia (EORTC QLQ-C30 insomnia score) scores corresponding to the time point at which cognitive impairment occurred were used in the logistic regression model. Using the median age as a cut-off, a subgroup analysis was conducted to evaluate whether similar genetic associations were observed in younger patients (below the median age of the cohort).

Results were reported as odds ratios (ORs) and 95% confidence intervals (CIs). Only cases with complete genetic and clinical information were included in the analysis. Statistical analyses were carried out using IBM’s Statistical Package for the Social Sciences (SPSS version 20) with *P* ≤ 0.05 considered as statistically significant.

## Results

### Patient demographics

**Figure 1:**
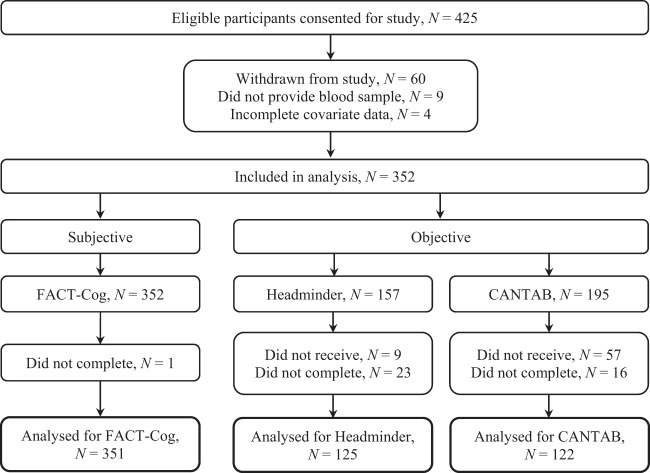
Study flow diagram.

A total of 425 patients were recruited, of which 351 patients were included in the final analysis (Fig. 1). The mean age (±SD) was 51.2 ± 9.1 years. Patients were predominantly Chinese (81.2%), and 85.2% had received at least high school education. Over half (59.8%) were diagnosed with stage II breast cancer and almost all (95.6%) had a baseline Eastern Cooperative Oncology Group (ECOG) score of 0, indicating an ambulatory status without restrictions on daily activities. Two-thirds (64.7%) of the patients received an anthracycline-based chemotherapy regimen. Among patients who completed objective neuropsychological tests, 125 (50.6%) were assessed using Headminder, while 122 (49.4%) were assessed using CANTAB. The demographic and clinical characteristics of the participants receiving Headminder and CANTAB were similar (Table 1). There was a statistically significant reduction in the FACT-Cog summation score as well as the domain scores over time (Supplementary Table S3) with approximately one-third of the patients experiencing clinically significant decline in self-perceived cognitive function (Table 2). Cognitive impairment was also observed in various objective cognitive domains, with incidences ranging from 7.2% to 36.9% (Table 2). Patients assessed with Headminder showed stable to improved cognitive function over time in 3 out of 4 cognitive domains. In contrast, improvement of cognitive function over time was only observed in 1 out of 4 cognitive domains among patients assessed with CANTAB (Supplementary Table S3).

**Table 1:** Demographic and clinical characteristics of the total cohort, Headminder participants and CANTAB participants.

Demographic and clinical information		Mean ± SD/Frequency, n (%)			p-value*
		Total (*N* = 351)	Headminder participants (*N* = 125)	CANTAB participants (*N* = 122)	
Age (years)		51.2 ± 9.1	49.9 ± 9.2	50.8 ± 8.6	0.40
Ethnicity	Chinese	285 (81.2)	100 (80.0)	99 (81.1)	0.86
	Malay	34 (9.7)	13 (10.4)	10 (8.2)	
	Indian	20 (5.7)	7 (5.6)	6 (4.9)	
	Others	12 (3.4)	5 (4.0)	7 (5.7)	
Education	No education	4 (1.1)	0 (0.0)	0 (0.0)	0.92
	Grade school	48 (13.7)	13 (10.4)	12 (9.8)	
	High school	160 (45.6)	53 (42.4)	55 (45.1)	
	Pre-university college	72 (20.5)	32 (25.6)	27 (22.1)	
	College/graduate degree	67 (19.1)	27 (21.6)	28 (23.0)	
Menopausal	Pre-menopausal	176 (50.1)	66 (52.8)	67 (54.9)	0.74
status	Post-menopausal	175 (49.9)	59 (47.2)	55 (45.1)	
Cancer stage	Stage I	61 (17.4)	30 (24.0)	19 (15.6)	0.94
	Stage II	210 (59.8)	62 (49.6)	80 (65.6)	
	Stage III	80 (22.8)	33 (26.4)	23 (18.9)	
ECOG status	0	336 (95.6)	117 (93.6)	119 (97.5)	0.13
	1	15 (4.3)	8 (6.4)	3 (2.5)	
Chemoregime	Anthracycline-based	227 (64.7)	78 (62.4)	79 (64.8)	0.7
	Taxane-based	124 (35.3)	47 (37.6)	43 (35.2)	
**Behavioral symptoms**					
Baseline fatigue (BFI total score)		1.6 ± 1.8	1.6 ± 1.7	1.8 ± 1.9	0.36
Baseline anxiety (BAI total score)		6.8 ± 6.7	6.7 ± 5.8	7.4 ± 8.3	0.49
Baseline insomnia score		22.7 ± 26.7	22.7 ± 26.0	24.3 ± 28.4	0.63

*Comparison between Headminder and CANTAB participants.

**Table 2:** Proportion of patients with CACI.

	Number of patients, n (%)		
	Overall*	At T2	At T3
**Impairment in subjective test measures** (**FACT-Cog**), ***N* = 351**			
Overall cognition	117 (33.3)	70 (19.9)	93 (26.5)
Mental acuity	103 (29.3)	57 (16.2)	84 (23.9)
Concentration	98 (27.9)	57 (16.2)	76 (21.7)
Multitasking	98 (27.9)	59 (16.8)	78 (22.2)
Verbal fluency	74 (21.1)	42 (12.0)	59 (16.8)
Memory	73 (20.8)	41 (11.7)	56 (16.0)
Functional interference	68 (19.4)	33 (9.4)	52 (14.8)
**Impairment in objective test measures** (**Headminder**), ***N* = 125**			
Learning and memory	21 (16.8)	12 (9.6)	16 (12.8)
Attention	20 (16.0)	14 (11.2)	9 (7.2)
Processing speed	13 (10.4)	7 (5.6)	7 (5.6)
Response speed	9 (7.2)	6 (4.8)	3 (2.4)
**Impairment in objective test measures** (**CANTAB**), ***N* = 122**			
Processing speed	45 (36.9)	26 (21.3)	35 (28.7)
Attention	32 (26.2)	18 (14.8)	25 (20.5)
Response speed	37 (30.3)	21 (17.2)	23 (18.9)
Learning and memory	17 (13.9)	12 (9.8)	8 (6.6)

^*^Impairment at either T2, T3 or both time points.

### Genotyping and allele frequencies

All patients included in the final analysis were successfully genotyped for *DNMT1* SNPs. A total of 215 (61.3%) patients were homozygous for the major allele (GG) of rs2162560 and 17 patients (4.8%) were homozygous for the minor allele (Table 3). A total of 153 patients (21.8%) were carriers of the A allele. There was no significant difference between the genotype frequencies between patients assessed by Headminder and CANTAB (*P* = 0.55).

**Table 3:** Genotype and allele frequencies of *DNMT1* rs2162560.

Genotype/Allele	Frequencies, n (%)		
	Total	Headminder participants	CANTAB participants
	(*N* = 351)	(*N* = 125)	(*N* = 122)
GG	215 (61.3)	80 (64.0)	70 (57.4)
GA	119 (33.9)	39 (31.2)	46 (37.7)
AA	17 (4.8)	6 (4.8)	6 (4.9)
G allele	549 (78.2)	199 (79.6)	186 (76.2)
A allele	153 (21.8)	51 (20.4)	58 (23.8)

### Association of DNMT1 SNP rs2162560 with CACI

Analysis of self-perceived cognitive impairment revealed that patients with the A allele of *DNMT1* rs2162560 had significantly lower odds of cognitive impairment in the concentration ability domain (OR = 0.45, 95% CI: 0.25–0.82, *P* = 0.01). Similarly, the presence of the A allele was also associated with lower odds of impairment in the functional interference domain (OR = 0.48, 95% CI: 0.24–0.95, *P* = 0.03) (Table 4). There was no statistically significant association between the *DNMT1* rs2162560 genotype and other cognitive domains. An analysis performed using a general genetic model yielded similar results (Supplementary Table S4).

**Table 4:** Association between *DNMT1* rs2162650 A allele and subjective CACI, *N* = 351.

Fact-cog domain	Odds ratio	*p* value	95% CI
Overall cognition	0.65	0.13	0.38–1.13
Mental acuity	0.70	0.22	0.39–1.24
Concentration	0.45	0.01^*^	0.25–0.82
Multi-tasking	0.99	0.97	0.56–1.73
Verbal fluency	0.68	0.23	0.37–1.27
Memory	0.69	0.23	0.37–1.27
Functional interference	0.48	0.03*	0.24–0.95

^*^P < 0.05.

No association between the *DNMT1* rs2162560 genotype and objective cognitive impairment assessed was observed (Table 5).

**Table 5:** Association between *DNMT1* rs2162650 A allele and objective CACI assessed by Headminder and CANTAB.

Cognitive domain	Headminder participants (*N* = 125)			CANTAB participants (*N* = 122)		
	Odds ratio	*p* value	95% CI	Odds ratio	*p* value	95% CI
Attention	0.82	0.72	0.28–2.44	0.97	0.95	0.40–2.37
Learning and memory	1.35	0.60	0.45–4.05	2.79	0.11	0.80–9.69
Processing speed	1.67	0.43	0.46–6.06	1.50	0.36	0.63–3.56
Response speed	1.42	0.65	0.32–6.42	1.33	0.51	0.58–3.06

### Subgroup analysis

A subgroup analysis was carried out with patients below the median age of the cohort, which was ≤51 years of age (*N* = 177). The results revealed that the *DNMT1* rs2162560 A allele was protective against deteriorations in the memory domain (OR = 0.26, 95% CI: 0.09–0.71, *P* = 0.01), concentration domain (OR = 0.30, 95% CI: 0.12–0.74, *P* = 0.01), and mental acuity domain (OR = 0.42, 95% CI = 0.18–0.96, *P* = 0.04) of the FACT-Cog (Supplementary Table S5).

## Discussion

In this study, we found an association between the *DNMT1* rs2162560 SNP and self-perceived cognitive impairment in breast cancer patients; carriers of the A allele experienced lower odds of self-reported cognitive decline in two cognitive domains: concentration and functional interference. These findings are relevant and novel as no known associations between SNPs in *DNMT1* and CACI have been described in the literature. A study involving 210 Brazilian Caucasian participants found no association between *DNMT1* rs2162560 and Alzheimer’s disease^19^. A study on 632 South Indians reported a significant association between schizophrenia and *DNMT1* rs2114724 and rs2228611, but no association was found with *DNMT1* rs2162560^20^. These results are not necessarily conflicting because cognitive disorders are complex and the mechanisms of disease manifestation in AD, schizophrenia, and CACI are very different. Both AD and CACI are associated with oxidative stress but they are propagated by different mechanistic pathways^32^. For example, subjective CACI has been characterized to be exacerbated by psychosocial or behavioral conditions including fatigue and anxiety^3^. In fact, the cognitive symptoms reported by patients are likely to be an extension of these conditions. The established association between *DNMT1* SNPs and behavioral disorders could explain why we observed an association between *DNMT1* rs2162560 and self-perceived cognitive impairment but not other domains of objective cognitive impairment. Furthermore, the populations analyzed in these studies may differ in genetic background and response to environmental factors, including chemotherapy treatment. DNA methylation is an epigenetic alteration that enables interaction with internal and external cues to create long-lasting changes in gene expression and possibly alter their homeostatic function and subjective experience^33^. Hence, the effects of *DNMT1* SNPs may vary according to disease state or population.

The rs2162560 polymorphism is located in the intronic region of the *DNMT1* gene on chromosome 19. Although introns are in the non-coding region, they may be involved in functions such as regulating alternative splicing or enhancing gene expression^34^. The rs2162560 mutant A allele is suspected to confer an intronic enhancer effect, similar to the *DNMT1* rs2114724 T allele, which is associated with schizophrenia^20^. Global methylation status, as measured by the methylation of surrogate marker LINE-1, is higher in men with at least one mutant rs2114724 allele^35^. Since rs2162560 and rs2114724 were highly correlated in a linkage disequilibrium study (coefficient = 0.85, *P* < 0.001)^15^, carriers of the rs2162560 polymorphism will likely display increased DNA methylation activity. This supports our hypothesis that the A allele of rs2162560 confers a protective effect in cognitive domains such as concentration and memory potentially by increasing methylation activity.

In cancer patients undergoing chemotherapy, both cancer and its treatment have been speculated to trigger reprogramming of the genome, resulting in changes to gene expression and neuronal transmission^33^. A study using the TumorGraft mouse model found that global DNA methylation and *DNMT1* levels decreased in the pre-frontal cortex of mice with triple-negative or progesterone-positive breast cancer TumorGrafts^36^. The A allele polymorphism may change *DNMT1* activity to mitigate these adverse effects.

Genome-wide DNA methylation and expression levels of DNMTs in the pre-frontal cortex and hippocampus decrease with age and affect learning and memory^37^. We found that the *DNMT1* rs2162560 A allele conferred a protective effect against declines in the memory, concentration, and mental acuity domains of the FACT-Cog in a younger subgroup of patients under 51 years of age. One possible explanation may be that, while rs2162560 exerts a protective effect through enhancing *DNMT1* function, the decreased amount of *DNMT1* in older patients diminishes any positive effect. Further studies are needed to elucidate how aging confounds the association between *DNMT1* polymorphisms and CACI.

This study has multiple strengths, including the pre-treatment and longitudinal assessment of cognitive function and behavioral symptoms across three time points, adjustment for known clinical confounders of cognition, and use of validated tools to evaluate cognition. We have also included both subjective and objective cognitive assessments, as recommended by the International Cognition and Cancer Task Force (ICCTF)^29^. Cognitive profiles observed in this study are highly consistent with our previous study where we have presented the existence of heterogeneous cognitive trajectories among cancer patients receiving chemotherapy^3^. As CACI is a multifactorial, complex phenotype, the precision of the results could be improved by adjusting for additional clinical, behavioral, and environmental factors, along with assessing different aspects of cognitive function. We adjusted for different covariates in the regression models (subjective and objective assessments) to ensure that the most clinically relevant variables were represented in the models. However, it remains unknown whether these results are applicable to delayed-onset CACI. Another limitation is changing the objective cognition assessment tool mid-study to CANTAB since the Headminder system was no longer commercially available. Due to this switch in assessment tools and other logistical issues, cognitive assessments were not completed for all participants. It is challenging to compare data generated from two different assessment tools, as they may have different sensitivities or subtle differences in the cognition functions assessed. This also explains why we observed fewer patients classified as impaired by Headminder than CANTAB. Hence, we reported the data obtained from both tools separately and did not pool the data. However, we ensured that all neuropsychological tests used were validated for the same cognitive domains^23,24^. We also calibrated the RCI to standardize the measurement of cognitive changes indicated by each tool.

## Conclusions

In conclusion, to the best of our knowledge, this is the first study to evaluate the genetic association between a *DNMT1* polymorphism and CACI. The A allele of rs2162560 showed a neuroprotective effect in the concentration and functional interference domains. These findings suggest that subjective CACI in cancer patients, which is often exacerbated by behavioral symptoms, is associated with epigenetic processes. Further validation of the current findings is required. These future studies should include measurement of plasma methylation levels at various time points to correlate the effect of *DNMT1* polymorphisms with cognitive function, which will provide further insight into the underlying epigenetic process. We could use this knowledge to identify patients who are at higher risk of developing CACI post-chemotherapy and provide targeted preventive strategies. Lastly, this study also provides the impetus to explore the use of pharmacological agents that target methylation in managing CACI.

## Supplementary information

Supplementary Data
