# Supplementary material for: An Evaluation of DNA Methyltransferase 1 (DNMT1) Single Nucleotide Polymorphisms and Chemotherapy-Associated Cognitive Impairment: A Prospective, Longitudinal Study
Source: Sci Rep. 2019 Oct 10;9:14570. doi: 10.1038/s41598-019-51203-y (PMC6787348; doi:10.1038/s41598-019-51203-y)
Supplement: Supplementary file 1 — Supplementary Data [file 41598_2019_51203_MOESM1_ESM.docx]

Research Article

An Evaluation of DNA Methyltransferase 1 (DNMT1) Single Nucleotide Polymorphism and Chemotherapy-Associated Cognitive Impairment: A Prospective, Longitudinal Study

Alexandre Chan^1,2,3^; Angie Yeo^1^, Maung Shwe^1^; Chia Jie Tan^1^; Koon Mian Foo^4^; Pat Chu^5^; Chiea Chuen Khor^6,7^; Han Kiat Ho^1^

^1^Department of Pharmacy, National University of Singapore, Singapore

^2^Department of Pharmacy, National Cancer Centre Singapore, Singapore

^3^Duke-N.U.S. Graduate Medical School Singapore, Singapore

^4^Department of Pharmacy, K.K. Women’s and Children’s Hospital, Singapore

^5^Singapore Cord Blood Bank, Singapore

^6^Human Genetics, Genome Institute of Singapore, Singapore

^7^Singapore Eye Research Institute, Singapore

This work was supported by the National University of Singapore [R-148-000-166-112, R-148-000-233-114]; the National Cancer Centre Singapore [NRFCB12131]; and the National Medical Research Council Singapore [NMRC/CIRG/1386/2014, NMRC/CIRG/1471/2017].

Corresponding Author:

Associate Professor Alexandre Chan

Department of Pharmacy, National University of Singapore

Block S4A Level 3, 18 Science Drive 4

Singapore 117543

Telephone: +65-6516-7814

Email: [phaac@nus.edu.sg](mailto:phaac@nus.edu.sg)

The authors declare no potential conflicts of interest.

This study was presented in part as an oral presentation at the 2016 American Society of Clinical Oncology (ASCO) meeting in Chicago, USA.

Keywords: DNMT1, epigenetic, rs2162560, breast cancer, cognition

Supplementary Table S1. List of objective test measures used

| **Domain** | **Measures** |
| --- | --- |
| **Headminder** | |
| Learning and memory | Memory Cabinet 1 (number correct), Memory Cabinet 2 (number correct), Incidental Learning 2 (number correct) |
| Attention | Number Recall (number correct), Number Sequencing (number correct) |
| Processing speed | Animal Decoding (number correct), Symbol Scanning (response time) |
| Response speed | Response Direction 1 (response time), Response Direction 2 (response time), Incidental Learning 1 (response time), Incidental Learning 2 (response time) |
| **CANTAB** | |
| Processing speed | Attention Switching Task/AST (mean corrected latency – non-switching blocks) |
| Attention | Rapid Visual Information Processing/RVP (mean latency) |
| Response speed | Reaction Time/RTI (five-choice reaction time) |
| Learning and memory | Paired Associates Learning/PAL (total errors – adjusted) |

Supplementary Table S2. Demographic characteristics of Headminder and CANTAB reference groups to facilitate the calculation of RCI for each tool

|  | | **Headminder** | **CANTAB** |
| --- | --- | --- | --- |
| **Demographic information** | | **(N=27)** | **(*N* = 17)** |
| Women |  | 27 (100) | 17 (100) |
| Age (years) |  | 51.7 ± 10.9 | 40.0 ± 11.3 |
| Ethnicity | Chinese  Malay  Indian  Others | 24 (85.7)  3 (10.7)  0 (0.0)  1 (3.6) | 10 (58.8)  2 (11.8)  3 (17.6)  2 (11.8) |
| Education | No education  Grade school  High school  Pre-university college  College / graduate degree | 0 (0)  5 (17.9)  11 (39.3)  6 (21.4)  6 (21.4) | 2 (11.8)  1 (5.9)  4 (23.5)  2 (11.8)  8 (47.1) |
| Menopausal status | Pre-menopausal  Post-menopausal | 14 (53.6)  13 (46.4) | 13 (76.5)  4 (23.5) |

Supplementary Table 3. Mean scores of cognitive test measures at each time point

|  | **Mean scores (SD)** | | | **p-value^a^** |
| --- | --- | --- | --- | --- |
|  | **T1** | **T2** | **T3** |  |
| **Subjective test measures (FACT-Cog^b^), *N* = 351** | | | | |
| Summation score  Mental acuity  Concentration  Multitasking  Verbal fluency  Memory  Functional interference | 132.7 (15.5)  14.4 (2.1)  14.3 (2.2)  13.8 (2.5)  22.0 (2.6)  24.6 (3.5)  14.3 (1.9) | 130.7 (19.1)  13.9 (2.7)  13.9 (2.6)  13.5 (2.8)  21.7 (3.2)  24.4 (3.8)  14.2 (2.2) | 127.9 (20.9)  13.4 (2.9)  13.5 (2.7)  13.2 (3.0)  21.3 (3.7)  23.7 (4.2)  13.9 (2.4) | <0.001*  <0.001*  <0.001*  <0.001*  0.002*  0.003*  0.152 |
| **Objective test measures (Headminder^b^), *N* = 125** | | | | |
| Learning and memory  Attention  Processing speed  Response speed | 103.4 (15.0)  97.4 (14.9)  104.3 (9.4)  105.2 (14.8) | 104.4 (16.1)  98.5 (15.9)  106.7 (7.0)  107.4 (14.6) | 102.1 (18.9)  100.6 (16.3)  107.5 (7.6)  107.4 (14.6) | 0.351  0.039*  <0.001*  0.047* |
| **Objective test measures (CANTAB^c^), *N* = 122** | | | | |
| Learning and memory  Attention (Mean latency)  Processing speed  Response speed | 18.8 (13.7)  401.3 (76.7)  618.5 (128.8)  320.2 (36.5) | 18.2 (24.1)  409.9 (80.9)  630.7 (121.2)  324.2 (36.1) | 14.9 (13.6)  414.9 (76.6)  639.5 (133.0)  325.6 (38.2) | <0.001*  0.318  0.161  0.180 |

^a^ p-values were generated using the Friedman test

^b^ Higher scores indicate better cognitive function

^c^ Higher scores indicate poorer cognitive function

* <0.05

Supplementary Table S4. Association between *DNMT1* rs2162650 A allele and subjective CACI, assuming a general genetic model, *N* = 351

| **Fact-Cog domain** | **Rs2162560 genotype** | **Odds ratio** | ***p* value** | **95% CI** |
| --- | --- | --- | --- | --- |
| Overall cognition | GA vs GG | 0.69 | 0.21 | 0.40-1.22 |
|  | AA vs GG | 0.40 | 0.21 | 0.10-1.68 |
| Mental acuity | GA vs GG | 0.71 | 0.26 | 0.39-1.29 |
|  | AA vs GG | 0.59 | 0.48 | 0.14-2.54 |
| Concentration | GA vs GG | 0.47 | 0.02* | 0.25-0.88 |
|  | AA vs GG | 0.33 | 0.18 | 0.06-1.67 |
| Multitasking | GA vs GG | 0.91 | 0.74 | 0.50-1.63 |
|  | AA vs GG | 1.72 | 0.38 | 0.52-5.73 |
| Verbal fluency | GA vs GG | 0.68 | 0.24 | 0.35-1.30 |
|  | AA vs GG | 0.75 | 0.69 | 0.18-3.15 |
| Memory | GA vs GG | 0.74 | 0.37 | 0.39-1.41 |
|  | AA vs GG | 0.37 | 0.24 | 0.07-1.95 |
| Functional interference | GA vs GG | 0.48 | 0.045* | 0.24-0.98 |
|  | AA vs GG | 0.47 | 0.37 | 0.09-2.47 |

**P < 0.05*

Supplementary Table S5. Association between *DNMT1* rs2162650 A allele and subjective CACI within the subgroup of patients aged ≤ 51 years (*N* = 177)

|  | **Odds ratio** | ***p* value** | **95% CI** |
| --- | --- | --- | --- |
| Overall cognition | 0.49 | 0.08 | 0.22-1.09 |
| Mental acuity | 0.42 | 0.04* | 0.18-0.96 |
| Concentration | 0.30 | 0.01* | 0.12-0.74 |
| Multi-tasking | 0.54 | 0.15 | 0.23-1.25 |
| Verbal fluency | 0.53 | 0.20 | 0.21-1.38 |
| Memory | 0.26 | 0.01* | 0.09-0.71 |
| Functional interference | 0.45 | 0.11 | 0.17-1.19 |

**P < 0.05*
